# Evaluation of Event-Based Algorithms for Optical Flow with Ground-Truth from Inertial Measurement Sensor

**DOI:** 10.3389/fnins.2016.00176

**Published:** 2016-04-25

**Authors:** Bodo Rueckauer, Tobi Delbruck

**Affiliations:** Institute of Neuroinformatics, University of Zurich and ETH ZurichZurich, Switzerland

**Keywords:** neuromorphic, AER, vision sensor, benchmarks, optical flow, inertial measurement unit, DVS, silicon retina

## Abstract

In this study we compare nine optical flow algorithms that locally measure the flow normal to edges according to accuracy and computation cost. In contrast to conventional, frame-based motion flow algorithms, our open-source implementations compute optical flow based on address-events from a neuromorphic Dynamic Vision Sensor (DVS). For this benchmarking we created a dataset of two synthesized and three real samples recorded from a 240 × 180 pixel Dynamic and Active-pixel Vision Sensor (DAVIS). This dataset contains events from the DVS as well as conventional frames to support testing state-of-the-art frame-based methods. We introduce a new source for the ground truth: In the special case that the perceived motion stems solely from a rotation of the vision sensor around its three camera axes, the true optical flow can be estimated using gyro data from the inertial measurement unit integrated with the DAVIS camera. This provides a ground-truth to which we can compare algorithms that measure optical flow by means of motion cues. An analysis of error sources led to the use of a refractory period, more accurate numerical derivatives and a Savitzky-Golay filter to achieve significant improvements in accuracy. Our pure Java implementations of two recently published algorithms reduce computational cost by up to 29% compared to the original implementations. Two of the algorithms introduced in this paper further speed up processing by a factor of 10 compared with the original implementations, at equal or better accuracy. On a desktop PC, they run in real-time on dense natural input recorded by a DAVIS camera.

## 1. Introduction

Accurate and fast measurement of optical flow is a necessary requirement for using this flow in vision tasks such as detecting moving obstacles crossing the path of a vehicle, visually guiding aircraft or space vehicle landing, or acquiring structure from motion information about the environment. The progress of optical flow estimation techniques is marked by two major stepping stones: The quantitative evaluation of optical flow algorithms by Barron et al. (1994) provided a dataset and error measures that became standard until succeeded by the more challenging Middlebury benchmark (Baker et al., 2010). Both helped identify difficult areas in flow estimation and opened the door for the development of dozens of new algorithms. Today, state-of-the-art techniques reach a remarkable degree of accuracy (see the evaluation on the Middlebury dataset online^1^). Barron et al. (1994) and Fleet and Weiss (2005) provide a basic introduction to gradient-based optical flow estimation; Sun et al. (2014) give an extensive review of current flow models. In spite of the large number of optical flow algorithms (116 on the Middlebury website at the time of submission), Sun et al. state that the majority of methods strongly resemble the original formulation of Horn and Schunck (1981). Their high accuracy requires massive computation and diminishes their usability in real-time applications (for instance, the highest-ranking algorithm on the Middlebury benchmark takes 11 min for two frames; the fastest has a runtime of 0.1 S.) However, with the development of asynchronous event-based artificial retinas (Posch et al., 2014; Liu et al., 2015) a promising new approach to visual signal processing has become possible.

In contrast to conventional image sensors, the dynamic vision sensor (DVS) camera produces not frames but asynchronous address-events (AE) as output (Lichtsteiner et al., 2008; Delbruck et al., 2010). They indicate positive and negative changes in log intensity at each pixel address, generating ON and OFF events respectively. This approach has several advantages: Encoding light with log intensity implements a form of local gain control, so the DVS can handle scene illuminations from a few lux to more than 100 klux, and in particular the local gain control enables reliable operation in scenes with high intrascene dynamic range. Events are conveyed to a processor over a standard USB interface at a temporal resolution of 1 μs with latency of at most 1 ms. The temporal resolution of the DVS is thus equivalent to that of a frame-based camera running at several thousand fps. This high sample rate is possible because redundant information originating from unchanged parts of the image are not transmitted, unlike in conventional cameras. This sparse AE output reduces memory requirements and computational cost and makes the DVS potentially useful for high-speed and low-power applications in robotics, avoidance of visual obstacles, terrain classification, etc.

Optical flow algorithms operating on the DVS output can benefit from these characteristics. They offer a solution to one challenge frame-based techniques face, namely large inter-frame displacements that occur in fast motion. For instance, Benosman et al. (2014) make use of the high temporal precision of DVS data by computing gradients on the surface consisting of most recent events. This method is explored in more detail in Section 2.1.3. Another problem of conventional optical flow methods is motion discontinuities at object boundaries, where at least two distinct motions overlap. The datasets offered in this paper do not have motion discontinuities, because the motions are generated by camera rotation. But in the case of DVS cameras, contrast edges at motion discontinuities would generate events exactly at the discontinuities, which Barranco et al. (2014) employs to extract the location and motion of contours. Furthermore, they combine DVS events with DAVIS intensity frames to reduce computational cost while at the same time increasing performance and stability. Away from strong contrast edges, in highly textured areas, event-based methods struggle because events that are fired during a short period of time at close-by positions are falsely assumed to stem from the same edge. Barranco et al. (2015) developed a phase-based method to improve estimates in textured regions. Brosch et al. (2015) provide a comprehensive analysis of event-based visual motion estimation. Furthermore, they suggest an event-based flow computation method using biologically inspired filter-banks that detect the orientation of an edge. A review of real-time bio-inspired visual motion estimation, particularly in hardware implementations, is given by Orchard and Etienne-Cummings (2014). They also propose a spiking neural network architecture which uses synaptic delay to create receptive fields sensitive to motion. All these developments are indicative of the potential of event-based techniques to resolve some of the major problems of conventional, frame-based flow estimation.

This work proposes a novel source of ground truth for optical flow evaluation, namely rate gyro data from an inertial measurement unit on the camera. In our dataset, camera motion is restricted to camera rotation, so all optical flow can by computed using the rate gyro information. A database is created and offered to compare several event-based optical flow algorithms. We introduce a simple smoothing filter to increase accuracy while reducing computation cost. The database as well as all the code is made public; the dataset link is provided together with a detailed description in Section 2.2 and each algorithm's open-source software implementation is provided in a footnote link. Section 3 presents the benchmarking results, which are discussed in Section 4 and lead us to the conclusion that economical real-time event-driven computation of motion flow is possible, but further development will be required for many practical applications.

## 2. Materials and methods

### 2.1. Event-based optical flow algorithms

We implemented and tested a direction selective filter (Delbruck, 2008), four variants of a basic Lucas-Kanade algorithm (Benosman et al., 2012), four variants of local plane fits (Benosman et al., 2014), and a flow estimation based on the camera's gyro information instead of visual motion cues. They are implemented in the open-source jAER project ^2^. jAER supplies infrastructure and algorithms for event-based sensor processing.

Except for the direction-selective (DS) method, this evaluation mainly focuses on gradient-based motion estimation methods that operate on the DVS AER events. Gradient-based approaches compute a first-order derivative on some image property to estimate motion. In frame-based methods, this property is typically luminance. While we refer to all methods in Sections 2.1.2 and 2.1.3 as gradient-based, note that they operate on a different function: The four Lucas-Kanade (LK) variants (Section 2.1.2) use a change in light intensity, while the four local-plane (LP) algorithms in Section 2.1.3 compute gradients on a surface containing the timestamps t(x,y) of the most recent DVS events as a function of pixel location.

#### 2.1.1. Direction selective filter

We refer to this method by the acronym “DS.” The DS method was developed by T. Delbruck in 2007 as the jAER class DirectionSelectiveFlow (https://sourceforge.net/p/jaer/code/HEAD/tree/jAER/trunk/src/ch/unizh/ini/jaer/projects/rbodo/opticalflow/DirectionSelectiveFlow.java) source code. This subclass of AbstractDirectionSelectiveFilter processes pure DVS event streams. Readers are referred to the source code for implementation details. DS was inspired by the organization of V1 direction selective simple cells (Delbruck, 2008, 2012).

In the DS algorithm, each incoming DVS event is preprocessed by an orientation filter called SimpleOrientationFilter^3^ in jAER. SimpleOrientationFilter first stores the current event's DVS timestamp in a 2D memory array indexed by pixel address. There are separate 2D arrays for ON and OFF DVS events. Next SimpleOrientationFilter checks the event's timestamp against the most recent DVS timestamps of its neighboring pixels along lines of pixels along 4 orientations (0, 45, 90, and 135°) to detect an orientated feature in the image. The number of pixels (length of the line) considered is a parameter which is typically 5 pixels. A moving edge produces nearly synchronous events along the edge. Thus, along the edge direction, the timestamp difference average between the current event and the most recent past DVS events will be minimized for the correct edge orientation. During this check, only events with sufficiently small timestamp difference are considered; if the past event is too old (typically more than 100 ms ago), then it is not counted. The orientation that is chosen for the edge is the one that produces the most synchronous activity; i.e., has the smallest average timestamp difference. The orientation event is labeled with the quantized orientation (at 0, 45, 90, or 135°). The orientation event timestamp (which is the same as the original DVS timestamp) is stored in a 2D array indexed by event address, again using separate arrays for each orientation and ON- and OFF-type edges, i.e., 8 orientation event arrays.

Next, for each orientation event, DirectionSelectiveFilter uses time of flight to compute the motion of this edge. It does this by computing the temporal interval of these orientation events to the most recent past orientation events along 2 lines of pixels extending out from the current event's pixel along the 2 directions perpendicular to the edge, i.e., if the edge is vertical, then the two horizontal directions left and right are checked. The distance is a parameter typically set to 5 pixels. The reciprocal of the average time difference between pixels is thus the speed of the edge in pixels per second. In this computation, past orientation event timestamps that are too old (typically more than 100 ms old) are not counted. In one of the two directions, the timestamp differences will likely indicate a reasonable speed; in the other direction the timestamp differences will typically be very large because the orientation events resulted from previous edges. The output events are labeled with this scalar speed and a quantized angular direction with values 0–7, 0 being upward motion, increasing by 45° counter-clockwise to 7 being motion up and to right.

#### 2.1.2. Lucas-Kanade variants

We refer to these methods by the acronym “LK.” They are implemented as the jAER class LukasKanedeFlow^4^. This algorithm is based on the method presented by Benosman et al. (2012). For each event, it uses a histogram of previous events in its neighborhood during a short time window to estimate a spatial and temporal gradient. This serves as input data to an overdetermined system of linear equations that can be solved for the optical flow vector with Least Squares Estimation.

The method makes use of the assumption that light intensity *I*(*x, y, t*) is invariant during an infinitesimally short time if we move along with the image with the correct optical flow. From this, the *gradient constraint equation*

(1) $$\nabla I^{T}\left[\begin{array}{c} v_{x}\\ v_{y} \end{array}\right]=-\frac{\partial I}{\partial t}.$$

can be derived using a Taylor series expansion (see e.g., Fleet and Weiss, 2005). Equation (1) simply points out that the time derivative is accounted for by the space derivative and flow. The velocity vector ${\left(v_{x},v_{y}\right)}^{T}$ is the motion flow we are looking for. But this single equation of two variables is under-determined, so we make a second (implicit) smoothness assumption: that the local flow ${\left(v_{x},v_{y}\right)}^{T}$ is constant over the *n*×*n* neighborhood of the pixel in question^5^. This leaves us with a system of *m* = *n*^2^ equations:

(2) $$\left[\begin{array}{c} \nabla I{\left(x_{1},y_{1}\right)}^{T}\\ \vdots \\ \nabla I{\left(x_{\text{m}},y_{\text{m}}\right)}^{T} \end{array}\right]\left[\begin{array}{c} v_{x}\\ v_{y} \end{array}\right]=\left[\begin{array}{c} -I_{t_{1}}\\ \vdots \\ -I_{t_{\text{m}}} \end{array}\right].$$

The Least-Squares solution to this matrix equation **Av** = **b** is given by **v** = (**A**^*T*^**A**)^−1^**A**^*T*^**b**. The covariance matrix **A**^*T*^**A** is invertible if its eigenvalues satisfy λ_1_ ≥ λ_2_ > 0. Thus, the eigenvalues serve as confidence measures, i.e., as means of determining the correctness of the computed velocities. No velocity is computed if both eigenvalues are smaller than a certain confidence threshold τ, i.e., λ_1_ < τ. If both are greater than τ, the matrix is considered invertible and the velocity can be computed as shown. If λ_1_ ≥ τ and λ_2_ < τ, we compute

(3) $$v=-I_{t}\frac{\nabla I}{\|\nabla I{\|}^{2}}.$$

Inserting Equation (3) back into the gradient constraint Equation (1) asserts the validity of this formula. Note that it is not feasible to use this equation in the first place, sidestepping the Least-Squares fit of the whole neighborhood. That would result in very noisy flow fields, while taking the whole neighborhood into account helps smooth local fluctuations.

The appropriate value of τ^6^ depends on the dataset; we found that values greater than 1 produce less noisy results.

##### 2.1.2.1. Backward finite difference

In the case of DVS output, the Equation (2) have to be reformulated in terms of address-events. To estimate the spatial gradients without gray levels (events are ON/OFF type only), one can count the number of events that happened in adjacent pixels in the neighborhood during the last Δ*t* μs. The difference in the number of events in neighboring pixels reflects relative changes in their brightness levels and thus provides an estimate of the local spatial gradient (See Benosman et al., 2012 for the full derivation). The *j*th row in the system of equations above then reads:

(4) $${\left[\begin{array}{c} \sum_{t-\Delta t}^{t}\left(e(x_{j},y_{j},t)-e(x_{j-1},y_{j},t)\right)\\ \sum_{t-\Delta t}^{t}\left(e(x_{j},y_{j},t)-e(x_{j},y_{j-1},t)\right) \end{array}\right]}^{T}\left[\begin{array}{c} v_{x}\\ v_{y} \end{array}\right]=\frac{1}{\Delta t}\sum_{t-\Delta t}^{t}e(x_{j},y_{j},t).$$

This equation relates the differential flow brightness consistency constraint to AER events. Image intensities are then approximated by event summations, because the dynamic vision sensor (DVS) does not provide absolute intensities. The results of this approach are shown in Section 3 under the label *LK*_*BD*_.

##### 2.1.2.2. Second order temporal derivative

As we will discuss in Section 4, the main problem of event-based gradient methods is the potentially small number of events in a neighborhood, making the derivative-estimation unstable. Brosch et al. (2015) show that the Lucas-Kanade method of Benosman et al. (2012) employs a mix of first and second derivatives. In short: The sum over events is itself a first order temporal derivative. This implies that the LHS of Equation (4) contains a temporal and spatial derivative, and the RHS should consequently contain a second order backward difference

(5) $$\begin{array}{l} {\left[\begin{array}{c} \sum_{t-\Delta t}^{t}\left(e(x_{j},y_{j},t)-e(x_{j-1},y_{j},t)\right)\\ \sum_{t-\Delta t}^{t}\left(e(x_{j},y_{j},t)-e(x_{j},y_{j-1},t)\right) \end{array}\right]}^{T}\left[\begin{array}{c} v_{x}\\ v_{y} \end{array}\right]=\\ \;\frac{1}{\Delta t}\left(\sum_{t-\Delta t}^{t}e(x_{j},y_{j},t)-\sum_{t-2\Delta t}^{t-\Delta t}e(x_{j},y_{j},t)\right). \end{array}$$

The effect on accuracy of this consistent use of second derivatives is discussed in Section 4. Note that one factor $\frac{1}{\Delta t}$ cancels out in Equation (4) because all terms contain one temporal derivative.

##### 2.1.2.3. Central finite difference (first order)

The original method Benosman et al. (2012) presented above utilizes a backward finite difference to estimate the gradient, i.e., the spatial derivative with respect to *x* is calculated by comparing the number of events at the pixel in question with those at the pixel one step to the left (one step down for the derivative with respect to *y*). We will show later that this asymmetric calculation clearly manifests itself in the optical flow vectors, which are biased toward the left and downwards. A symmetric gradient calculation (*e*(*x*_*j*+1_, *y*_*j*_, *t*)−*e*(*x*_*j*−1_, *y*_*j*_, *t*))/2 and (*e*(*x*_*j*_, *y*_*j*+1_, *t*)−*e*(*x*_*j*_, *y*_*j*−1_, *t*))/2 removes this bias. We evaluate the performance of this variation *LK*_*CD*1_ in Section 3.

##### 2.1.2.4. Central finite difference (second order)

Because of the crucial role the derivative approximation plays in estimating flow vectors here, we tried the effect of applying a finite difference with higher order of accuracy by using more points on the accumulated event histogram. Above, the central difference coefficients are (−1/2, 0, 1/2) at locations (*j*−1, *j, j*+1). The next higher accuracy is achieved with coefficients (1/12, −2/3, 0, 2/3, −1/12) at locations (*j*−2, *j*−1, *j, j*+1, *j*+2). We label this variation of the Lucas-Kanade method *LK*_*CD*2_ and compare it in Section 3.

##### 2.1.2.5. Savitzky-Golay filter

The event-based Lucas-Kanade method treated here computes derivatives of an *event rate function* that counts the number of events that occurred in a certain time window in the past. This digital event rate function usually takes on small values and is quite susceptible to noise. One way to increase the signal-to-noise ratio is the Savitzky-Golay filter (SG), which is introduced here in the context of the Lucas-Kanade method as well as being used later in the Local Planes method described in Section 2.1.3. This digital filter convolves data by fitting a low-order polynomial to adjacent points with linear least squares. From the first order fit coefficients, the gradients of the fitted surface are then immediately available. It requires the measurement to be at the center, a symmetric weighting, a rectangular grid and inclusion of all grid points (Thornley, 2006). The two-dimensional function is approximated by the polynomial

(6) $$f(x,y):=\sum_{p=0}^{n}\sum_{q=0}^{n-p}a_{pq}x^{p}y^{q}$$

where *n* gives the degree of the polynomial in *x*- and *y*-direction (we confine ourselves to polynomials of the same degree in both directions). The coefficient matrix **a** is determined in a least-squares fit of the model to the data points $\text{d}={\left(t\left(x_{i},y_{i}\right)\right)}^{T}$, 1 ≤ *i* ≤ *m* in the neighborhood:

(7) a=Cd

with the pseudo-inverse **C** = (**B**^*T*^**B**)^−1^**B**^*T*^ of the matrix containing the polynomial terms ${\left(\text{B}\right)}_{pq}=x^{p}y^{q}$. The vector **d** contains the neighborhood points, e.g., in the case of a 3 × 3 neighborhood, **d** is a 9-vector where the first three elements are the top row of pixels, etc. Then C is a 3 × 9 matrix that computes the 3 components of **a** = {*a*_00_, *a*_10_, *a*_01_}. Thus, **C** is a set of three 3 × 3 kernels {*c*_00_, *c*_10_, *c*_01_} that act on the pixel neighborhood, where each of kernels is a row of **C**.

For the specific example of a first order filter that estimates the first derivatives in a 3 × 3 neighborhood, Equation (7) results in kernels in {*c*_10_, *c*_01_} (rearranged to show the spatial operation on the neighborhood)

$$c_{01}=\left(\begin{array}{ccc} +\frac{1}{6} & +\frac{1}{6} & +\frac{1}{6}\\ 0 & 0 & 0\\ -\frac{1}{6} & -\frac{1}{6} & -\frac{1}{6} \end{array}\right),\;c_{10}=\left(\begin{array}{ccc} -\frac{1}{6} & 0 & +\frac{1}{6}\\ -\frac{1}{6} & 0 & +\frac{1}{6}\\ -\frac{1}{6} & 0 & +\frac{1}{6} \end{array}\right)$$

where it should be clear that these kernels estimate the first derivatives in y- and x-directions as averages in the perpendicular x- and y-directions.

The convolution coefficients **C** need only be calculated once for a certain polynomial order and neighborhood size. A 2D polynomial fit of degree (*n, n*) uses *k* = (*n* + 1)(*n* + 2)/2 parameters (e.g., *k* = 3 for a linear 2D fit), so the neighborhood must consist of *m* ≥ *k* pixels. To obtain the smoothed event rate function, the *k* fit parameters *a*_*pq*_ are calculated once for the neighborhood of the event in question, using Equation (7). Now the gradient of this surface can be read out directly from the first order fit terms. It is not necessary to evaluate the fitted function over all the points, but these smoothed points could be computed by inserting each x,y coordinate of the neighborhood into Equation (6). The results of using the SG filter are presented in Section 3 under the label *LK*_*SG*_.

#### 2.1.3. Local plane fits

We refer to these methods by the acronym “LP.” They are implemented as the jAER class LocalPlanesFlow^7^. This algorithm is based on the method presented in Benosman et al. (2014). It uses the local properties of events' spatio-temporal neighborhood by fitting a plane to an incoming event's neighborhood on the surface of recent events. Precise visual flow orientation and amplitude can be estimated using a local differential approach on the surface defined by recent events. In contrast to the methods above, this does not necessitate estimation of spatial and temporal gradients.

When each event at pixel location (*x, y*) coming from the DVS at time *t* is drawn into a 3d coordinate system (and previous events at the same location discarded), we obtain the surface of active events. In analogy to the algorithms in Section 2.1.2, we add a regularization process by assuming constant local velocity in a small neighborhood of the event. This makes the flow estimation robust against noise and compensates for missing events in the neighborhood of active events where motion is being computed. In our visualization of events as a surface, this local velocity constancy corresponds to the neighborhood being locally planar. The fitting parameters (*a, b, c, d*) of such a local plane *ax* + *by* + *ct* + *d* = 0 are determined by solving a homogeneous system of equations with least squares regression. The LocalPlanes algorithm includes an iterative improvement of this initial plane fit, where events are discarded that are further away from the plane than a certain threshold timestamp difference th2 (typically 10 ms). The least squares fit is then repeated with the remaining points. This continues until the square root of the summed differences between the current fit parameters (*a, b, c, d*) and the previous ones drops below a second threshold th1 (typically 0.01). With the parameters obtained like this, the velocity is given by the inverse gradient:

(8) $$\left[\begin{array}{c} v_{x}\\ v_{y} \end{array}\right]=-c\left[\begin{array}{c} \frac{1}{a}\\ \frac{1}{b} \end{array}\right].$$

This formulation assumes time to be a strictly increasing function of space, such that the local derivatives *a, b* are never zero. In practice, these derivatives are often zero or very small. When the DVS records a moving edge, it fires a line of events with similar timestamp. This line is problematic in the special cases where the edge is oriented approximately along the x- or y-axis. The gradient of the resulting local plane will have a vanishing component along the edge orientation. The equations above interpret the situation falsely as infinitely fast motion along the edge, though there really is none. To avoid inverting these vanishing gradients, the velocity components corresponding to derivatives with magnitude below a certain threshold (e.g., th3) can be set to zero. This results in angular quantization: For instance, the y-components of flow vectors of a rotating edge with orientation close to the y-axis are truncated to zero, because the y-derivatives are close to zero. Thus, the flow vectors resulting from Eq 8 are correct only when the edge orientation is far enough from being vertical or horizontal.

To correctly deal with vanishing gradients in one direction, we first use the fact that the true *direction* of motion is encoded by the gradient $\text{g}=\left(-\frac{a}{c},-\frac{b}{c}\right)$ of the fitted plane. But because its components describe the change of time with respect to space, the *magnitude* (and units) does not match. We therefore normalize this gradient vector and then multiply it by the correct length, which is given by the inverse of its magnitude: $|\text{g}|=\sqrt{a^{2}+b^{2}}/c$. The velocity vector is thus obtained as

(9) $$\left[\begin{array}{c} v_{x}\\ v_{y} \end{array}\right]=\frac{1}{|g{|}^{2}}g=\frac{-c}{a^{2}+b^{2}}\left[\begin{array}{c} a\\ b \end{array}\right].$$

This formulation is robust against vanishing derivatives *a* or *b*.

As a confidence measure for the rare case that both gradients vanish (flat plane, infinite velocity), we introduce a threshold th3. If both *a* and *b* are below that value, the computed velocity is interpreted as unrealistically high and discarded. The optimal value depends on the application; for the present dataset (which does not contain motions at high speed) we chose th3 of about 1e-3; because the units of time are microseconds, speeds in excess of 10^3^ pixels/second are not reported.

##### 2.1.3.1. Orthogonality constraint

Brosch et al. (2015) proposed another way to remedy the problem described above, namely that the surface function is not injective and thus not invertible when timestamps are similar along an edge. They derive the exact same formula for the velocity vector $\text{v}={\left(v_{x},v_{y}\right)}^{T}$ by arguing that the plane's normal vector **n** = (*a, b, c*)^*T*^, the contour orientation $\text{l}={\left(l_{x},l_{y},0\right)}^{T}$ and the velocity vector ${\text{v}}_{h}={\left(v_{x},v_{y},1\right)}^{T}$ defined in homogeneous coordinates are mutually orthogonal. Solving the three equations **n**^*T*^**u** = 0, **n**^*T*^**l** = 0 and **u**^*T*^**l** = 0 for *v*_*x*_, *v*_*y*_ results in Equation (9). However, using homogeneous coordinates in this way is misleading, as it implies checking orthogonality between vectors of different vector spaces: The normal **n** is part of euclidean ℝ^3^ (with scale c rather than 1 as in **v**_*h*_), whereas $\text{l}={\left(l_{x},l_{y},0\right)}^{T}$ interpreted in homogeneous coordinates represents a point at infinity. Fortunately, Equation (9) can be obtained staying purely in ℝ^3^. Instead of **v**_*h*_, we use the vector containing infinitesimal increments along the x, y, and t axis: $\text{u}={\left(dx,dy,dt\right)}^{T}=dt{\left(\frac{dx}{dt},\frac{dy}{dt},1\right)}^{T}=dt{\left(v_{x},v_{y},1\right)}^{T}$. This vector is embedded in the plane and perpendicular to both the normal and the edge orientation. It is clearly equivalent to **v**_*h*_ up to a scale dt, which drops out in the orthogonality relations. Thus, we arrive at the same result without the need to change over to projective space.

We implemented both the original method^8^ (using Equation 8) and the robust formulation^9^ (Equation 9). The results are listed in Section 3; the methods are labeled *LP*_*orig*_ and *LP*_*robust*_ respectively.

##### 2.1.3.2. Single fit

How critical for accuracy is the iterative improvement of the plane fit, as described in the second paragraph of this section? One could argue that the iterative rejection of distant events makes sense only when the fit is already good: If the initial plane happened to misrepresent local motion, a valid event would be considered an outlier and wrongly removed. Using simply the initial fit to estimate the gradient substantially reduces the amount of computations needed: The re-computation of the plane equation for the whole neighborhood as well as the eigenvalue-decomposition for the reduced data matrix drop out. But even for the initial plane fit we can dispense with the costly eigenvalue-decomposition. The vertical offset *d* of the plane *ax* + *by* + *ct* + *d* = 0 contains no information about the gradient and therefore does not contribute directly to the optical flow estimation. It *is* needed when checking the distance of an event to the plane during iterative improvement. But when we confine ourselves to the initial fit, we can divide the plane equation by *d* and rearrange it to $ãx+\tilde{b}y+\tilde{c}t=-1$. Taking into account the whole neighborhood, this now gives us an inhomogeneous system of equations in three variables and can be solved inexpensively with the pseudo-inverse.

We measured the difference in processing time for both methods as well as the error right after the initial fit (labeled *LP*_*SF*_)^10^ to see if we can achieve comparably good accuracy with this simplification. The result is shown in Section 3.

##### 2.1.3.3. Savitzky-Golay filter

In an attempt to smooth the often noisy surface of recent events without distorting the signal too much, we applied a two-dimensional linear Savitzky-Golay filter as described above. The same as for the event rate function, we could obtain the smoothed surface by calculating the *k* fit parameters *a*_*pq*_ once for the neighborhood of the event using Equation (7) and inserting each point of the neighborhood into Equation (6). However, if interested only in the gradient of a linear fit as in the context of the Local Planes method, this last step can be skipped. After computing the three parameters ${\left(a_{00},a_{01},a_{10}\right)}^{T}$ of a first-order Savitzky-Golay filter, the flow vector is given by Equation (9), where *c* = −1 to map the Savitzky-Golay fit *f*(*x, y*) = *a*_00_+*a*_10_*x*+*a*_01_*y* to the plane equation *ax*+*by*+*ct*+*d* = 0.

Computing the parameters **a** with Equation (7) assumes a complete grid of valid data points. Due to the sparse nature of DVS events, most of the time this completeness is not the case: Locations in the neighborhood may contain old events; the event surface ahead of a leading edge consists of either unset timestamps or old events belonging to objects passing previously. Equation (7) offers no way to discard them robustly. The solution is to perform the low-level operations that are contained in the first-order Savitzky-Golay kernels **C** one by one, namely estimating the average derivatives in X and Y of the timestamp surface, taking account of only valid timestamps. This robust estimation of the slopes iterates over the rows (for X-derivative) or columns (for Y-derivative) of the timestamp array and computes the finite differences for valid pairs of points. The final gradient in X and Y is the average of these individual derivative estimates. It can be shown that this algorithm is equivalent to applying a linear Savitzky-Golay fit - with the benefit of robustness against invalid data points, at the cost of more computations.

The results of this method are shown in Section 3 under the label *LP*_*SG*_^11^.

#### 2.1.4. Inertial measurement unit (IMU)

We refer to this method of computing optical flow as IMU. It is implemented as the jAER class IMUFlow^12^. The DAVIS240C camera^13^ we used in our experiments includes an IMU (Inertial Measurement Unit) that provides gyro and accelerometer data, i.e., the sensors for an electronic vestibular system (Delbruck et al., 2014). Here we used only the rate gyro information. The pure camera rotation used in this study allows us to compute the ground truth true optical flow (the motion field) from the IMU gyro rates, as follows.

**Figure 1:**
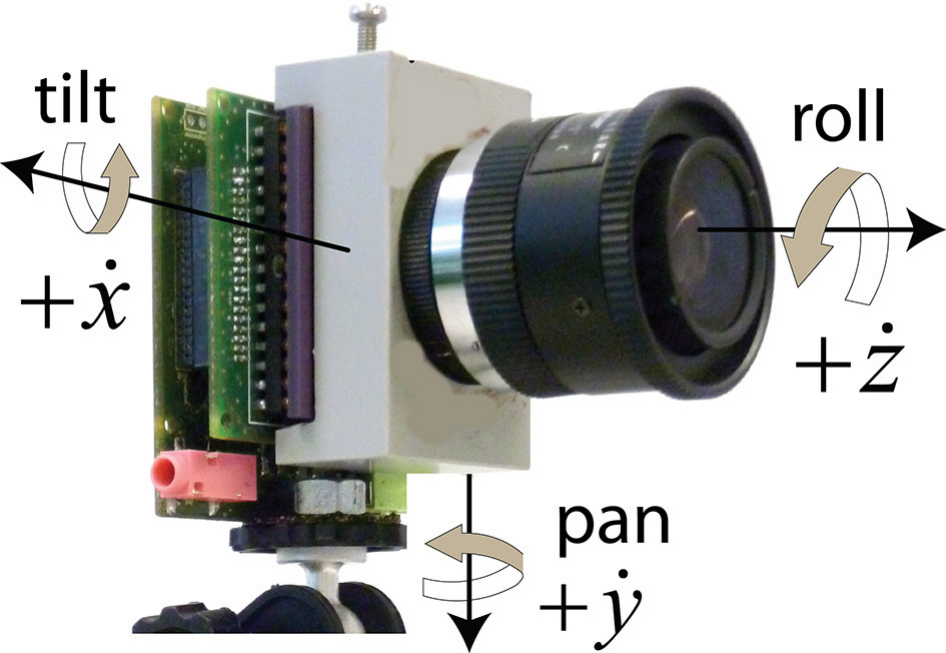
**The rate gyro axes of a DAVIS240 camera including an IMU**. (Graphic adapted from Delbruck et al., 2014.)

The gyro data is given in rotational angular rates around the three camera axes (see Figure 1). The angular rates ẋ, ẏ, ż stand for tilt (up/down), pan (right/left) and roll (camera axis rotation). To obtain total camera rotational angles *x, y, z*, these angular rates are integrated over time and high-pass filtered to deal with gyro offsets which were sometimes as large as 2°/s. The first-order IIR filter with time constant τ (set to 10 s in this study) computes an integrated angle *x*_*n*_ from an IMU sample ẋ_*n*_ taken at time *t*_*n*_ from Equation (10):

(10) $$x_{\text{n}}={\dot{x}}_{\text{n}}\Delta t_{\text{n}}+(1-\frac{\Delta t_{\text{n}}}{\tau })x_{\text{n-1}}$$

(11) $$\Delta t_{\text{n}}=t_{\text{n}}-t_{\text{n-1}}.$$

The subscript n indicates the position in the sample series (we drop it in the following). The IMU updates the angular rates at a sample rate of 2.5 kHz (the IMU was fully interfaced to the camera CPLD since publication of Delbruck et al., 2014). When any of the *x, y, z* values exceed a certain limit (typically 45°), then all are set back to zero. This reset is important during continued camera rotation and in effect implements saccades to re-center the transformed output.

**Figure 2:**
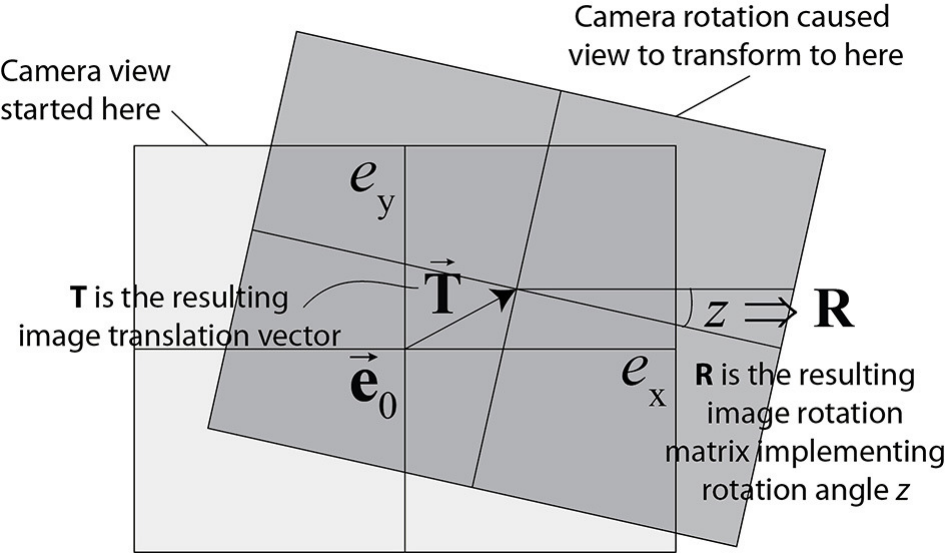
**Transformation of coordinates due to camera rotation**. Lens distortion is neglected (pinhole model; rectangle conserves its shape). This assumption works well for lenses with large focal length. (Graphic adapted from Delbruck et al., 2014.)

These rates offer way to obtain an estimate of true optical flow produced by a pure rotation of the camera. As shown in Delbruck et al. (2014), the rotation of the camera transforms a DVS event address **e** = (*e*_*x*_, *e*_*y*_) according to Equation (12):

(12) $$e\prime =R(e-e_{0}+T)+e_{0}$$

where the 2-vector **e**′ is the transformed event address, **e**_0_ is the pixel address nearest the center of the IMU, **R** is the 2 × 2 roll-angle image rotation matrix, and the 2-vector **T** is the translation of the image due to pan and tilt rotations. An illustration of how an event is rotated and translated is given in Figure 2. The DAVIS240C camera has the IMU mounted directly behind and centered under the image sensor pixel array center at a distance of about 3 mm from it, so in practice *e*_0_ is the center of the pixel array. Equation (12) assumes a pinhole lens with no distortion, so that all pixels have the same magnification, which was approximately the case for the lens used in this study, although on the periphery, some slight lens distortion is evident from the data in **Figure 4** for the translating sinusoid, where the bars are slightly curved on the corners of the image (see Section 2.2.2).

The z image rotation matrix **R** and x-y image translation vector **T** are given by Equation (13):

(13) $$T=k\left[\begin{array}{c} y\\ x \end{array}\right],\;R=\left[\begin{array}{cc} \cos z & -\sin z\\ \sin z & \;\cos z \end{array}\right]$$

To scale from camera pan and tilt rotation angle to DVS pixels, we multiply with the conversion factor

(14) $$k=\frac{1}{\tan^{-1}(\frac{w}{l})}.$$

This equation is derived from the trigonometric identity that the tangent of the angle that subtends one pixel is equal to the pixel pitch *w* divided by the lens focal length *l*. For the camera used here, *w* = 18.5μm and *l* = 4.5*mm* (Kowa, f/1.4 C-mount lens), thus *k* = 4.25 pixels/°.

Once we know the transformed event's address **e**′ we obtain the local optical flow of the motion field from the difference in coordinates Δ**r** = **e**′ − **e** from Equation (12), and the velocity **v**(*e*) = Δ**r**/Δ*t* by dividing by the time since the last IMU gyro rate update.

This computation of course assumes that the objects seen by the camera are stationary, and the camera is only rotating, not translating through space. In this study, we excluded camera translations by fixing the sensor on a rotor (see Section 2.2).

The implemented computation of the motion field based on integration and then discrete differentiation of the gyro rates for each event is inherited from the dual use in jAER of the rate gyro information for stabilizing (derotating) the DVS output (Delbruck et al., 2014). A direct computation of the motion field from the gyro rates is also possible.

Optical flow estimated from IMU gyro data is useful as ground truth where either the visual motion field (not just normal flow) is measured, or the orientation of the contour is known so that the ground truth vectors can be projected onto its normal (e.g., in direction selective filters).

### 2.2. Dataset

To evaluate the methods presented above, we created a public dataset^14^ consisting of two synthesized samples, two real samples with simple structure, and one real, more complex indoor scene. We have also included DAVIS data and an extracted image of a standard checkerboard pattern useful for camera calibration in the dataset. An illustrated online README document provides a guide to the dataset, examples of using it, and a step-by-step guide to jAER software installation and flow algorithm use. The real samples were recorded from a 240 × 180 pixel DAVIS240c (Dynamic and Active-pixel Vision Sensor, Brandli et al., 2014). This vision sensor combines the functionality of a DVS with frame-based absolute intensity read-out^15^. Thus, the recorded dataset contains asynchronous events from the DVS as well as conventional synchronous frames to support testing state-of-the-art frame-based methods. The frames are not used in this study.

Note that the gradient-based and direction-selective methods discussed here only compute normal flow, i.e., the velocity perpendicular to the orientation of a moving edge. We therefore focused on collecting a dataset that contained only normal flow.

#### 2.2.1. Synthesized samples

Address-events with timestamp and polarity can be created in Matlab and imported into jAER. The script for creating this dataset is available in the dataset and the script for importing these synthetic events is located in the jAER repository in the matlab folder^16^. In the simplest case, such samples are clean signals without occlusion, specularity, shadowing, transparency etc. Furthermore, the ground truth is known from the underlying model. The early benchmarking dataset by Barron et al. (1994) contained two such plain samples. Our synthesized samples tie in with them and cannot be compared to the Middlebury artificial samples with respect to complexity. We believe that these elementary datasets are still justified in testing the relatively young branch of event-based flow estimation. We will see in Section 3 that we cannot count on high accuracy even for relatively simple samples. In any case they are a useful tool to check that a new implementation works in principle.

##### 2.2.1.1. Translating square

The first is a square of width 40 pixels translating with *v* = (20, 20) pixels/s. The events are concentrated solely along the edges, so this corresponds to an texture-less object with perfect contours. This sample demonstrates how the algorithms output normal flow rather than object flow (here: along the horizontal and vertical axis rather than diagonally toward the upper right corner).

##### 2.2.1.2. Rotating bar

The second sample is a bar of width 1 pixel and length 50 pixels rotating with rotation rate 0.21 Hz. In this case, normal flow matches object flow. We expect the flow vector direction to vary continuously with the rotation angle, and the speed to be proportional to the distance from the center of rotation.

#### 2.2.2. Real samples

To record the samples from real input, the DVS was mounted on a rotor which restricted motion to either pan, tilt or roll, depending on how the camera was fastened. The pan, tilt and roll were arranged to create pure camera rotation; in the case of pan and tilt movements the scene was sufficiently far from sensor so that the slight camera translation created negligible flow. The rotational speed was controlled via a DC-voltage source. The visual stimulus for the rotating bar and translating sinusoid was printed on paper and fixed in front of the camera at a distance of approximately 30 cm. Images of the experimental setup and of the input stimuli are provided in the supplementary material.

##### 2.2.2.1. Translating sinusoid

The first real sample shows a contrast pattern that varies sinusoidally in horizontal direction. The camera is panned clockwise around its y-axis to create the impression that the sinusoid is shifting to the left. The contours are slightly curved due to lens distortion. The consequence is that the ground truth from the IMU is not perfectly normal everywhere on the contours. This introduces a systematic error of maximum 2.2 pixels or 8° at the corners of the image in the flow vectors on the periphery of the image. Such distortions can be corrected by the fully-integrated *SingleCameraCalibration* camera calibration method, data for which is available in the dataset.

##### 2.2.2.2. Rotating disk

The second sample contains a disk with eight compartments of varying gray-levels. The camera is rotated around its z-axis (roll) so the disk seems to turn clockwise.

##### 2.2.2.3. Translating boxes

The third sample shows a table with two cases and books. The scene contains some shadows, reflective components, specularities, parts with little contrast between fore- and background, no highly textured surface, no complex motion discontinuities, no transparency. The motion is purely translational and caused only by the panning camera.

#### 2.2.3. Ground truth

In their concluding remarks, Barranco et al. (2014) call for the creation of new benchmarks with ground truth for neuromorphic cameras. There is a variety of approaches to obtaining ground truth. For synthesized sequences, the true motion field can be constructed from geometric principles or is known from the underlying model used to generate the sample. Except for such synthesized datasets, the ground truth is itself the result of measurements and therefore to some degree imperfect. In Barranco et al. (2015), the authors mount a camera on a robot and use odometry and depth-measurements to collect the ground truth. In Baker et al. (2010), they spray a fine grid of phosphoric substance onto the scene and measure the displacements with UV light. Another possibility to obtain the ground truth for event-based flow evaluation is to interpolate frame-based datasets (Barranco et al., 2014, 2015) like the Middlebury dataset, where the ground truth is provided. We could not make use of this option because the methods evaluated here produce only normal flow, and to get meaningful error statistics the ground truth should also be normal to contours. In Barranco et al. (2015), the ground truth (derived from the Middlebury dataset as mentioned above) is projected onto the gradient direction computed by their event-based contour estimation. This way they can compare their measured normal flow to the normal ground truth.

As a novel source of ground truth we propose using the flow field obtained from IMU gyro data as explained in Section 2.1.4. When using a camera that hosts an inertial measurement unit, the ground truth can be calculated in real time and parallel to any other filters applied to the incoming frames or events. Optical flow measurements based on visual cues can be compared directly to the IMU flow, which can serve as feedback or initial guess and to discard outliers. It has some obvious limitations, however: the flow field can only be calculated from the IMU, if the camera is rotating about its axes. Also, the scene has to be static; objects moving independently in the scene would be labeled with the same IMU-deduced flow as resting objects. Finally, there is no depth distinction. For these reasons the dataset created here does not contain moving objects or non-rigid motion.

### 2.3. Error measures

#### 2.3.1. Average endpoint error (AEE)

We use the established absolute Average Endpoint Error

(15) $$\text{AEE}=\frac{1}{N}\sum_{i=1}^{N}\sqrt{{\left(v_{x,i}-u_{x,i}\right)}^{2}+{\left(v_{y,i}-u_{y,i}\right)}^{2}}$$

as well as the relative AEE, normalized with respect to the ground truth speed,

(16) $${\text{AEE}}_{\text{rel}}=\frac{1}{N}\sum_{i=1}^{N}\sqrt{{\left(v_{x,i}-u_{x,i}\right)}^{2}+{\left(v_{y,i}-u_{y,i}\right)}^{2}}\frac{1}{\left|u_{i}\right|}$$

where the vector **v**_*i*_ = (_*v*_*x*_, *v*_*y*_)*i*_ stands for the i-th sample of measured flow and **u**_*i*_ = (_*u*_*x*_, *u*_*y*_)*i*_ for the corresponding ground truth flow vector.

#### 2.3.2. Average angular error (AAE)

The endpoint error does not distinguish between angular deviation and speed difference. Therefore, we measure the angular error as well. In the frame-based optical flow literature, the angular error is not purely angular in the image plane, because it considers the angle in space-time (pixel,pixel,frame), i.e., by computing it as (Barron et al., 1994)

(17) $${\text{AE}}_{\text{3D}}=\arccos\frac{1+v_{x}u_{x}+v_{y}u_{y}}{\sqrt{1+v_{x}^{2}+v_{y}^{2}}\sqrt{1+u_{x}^{2}+u_{y}^{2}}}.$$

This angular error is between vectors in 3D with a constant third component as arbitrary scaling constant that also prevents division by zero. To see how this measure combines angular and length error, compare the angular error between velocity vectors (1,0) and (2,0): The angular error in the image plane is zero, the endpoint error is not and neither is Equation (17). The additional third component in (*v*_*x*_, *v*_*y*_, 1), i.e., the combination of angular error in the 2D image plane and length error, causes this bias that has been pointed out in Barron et al. (1994) and Baker et al. (2010), which leads most authors to favor the AEE. We consider a purely angular error metric between vectors in the image plane beneficial, as it helps identify the specific shortcomings of an implementation. Thus, to separate AE and EE, we compute the Average Angular Error between the velocity vectors in the image plane:

(18) $$\text{AAE}=\frac{1}{N}\sum_{i=1}^{N}\arccos\frac{v_{x,i}u_{x,i}+v_{y,i}u_{y,i}}{\left|v_{i}||u_{i}\right|}.$$

Zero-velocity measurements are not counted in the computation of Equation (18).

### 2.4. Statistics

We display the Standard Deviation (SD) instead of the Standard Error of the mean (SE) because we are interested in the spread of the data points around the mean rather than the closeness of the sample mean to the population mean.

In addition to the average and SD, we report similar robustness measures as in Baker et al. (2010): Rx is the percentage of pixels that have an error measure above × (e.g., R2.5 in degrees for angular error and R2 in pixels for endpoint error). However, we use higher values than the Middlebury evaluation because the error histograms are considerably wider.

### 2.5. Processing time measurement

The computation time of an optical flow vector for a single event is usually smaller than the resolution of the time measurement function used (e.g., Java's System.nanoTime() has a resolution of microseconds on our Windows computer). The events have to be buffered into event packets and the total computation time then averaged. However, the algorithms discard events during computation of flow. Not all events can be used: for instance, the neighborhood of an event may be so sparsely populated that the data matrix used in the Least-Squares Regression is singular. Then no motion vector is calculated. Other tests include a refractory period or a speed control. Dividing the total processing time of the packet by the initial input size would result in a mean processing time which could be several orders of magnitude too small, because not all events actually make it all the way through the filter. To circumvent this, all unnecessary filters (like refractory period and speed control) are turned off; in those tests that are inherent to the flow computation (like invertibility), the event is assigned zero velocity and continued to be processed normally, so that no regular part of the filter is skipped. Another option could be to filter out in a first stage all the events that would produce invalid results, and in a second stage measure the computation time for the remaining set with known size.

The real-time cost of processing an event was measured on a Core i5 @ 2.4 GHz PC running Windows 10 × 64 and Java 1.7. The overhead on this machine simply to process event packets is of the order of tens ns/event and can be neglected.

## 3. Results

**Figure 3:**
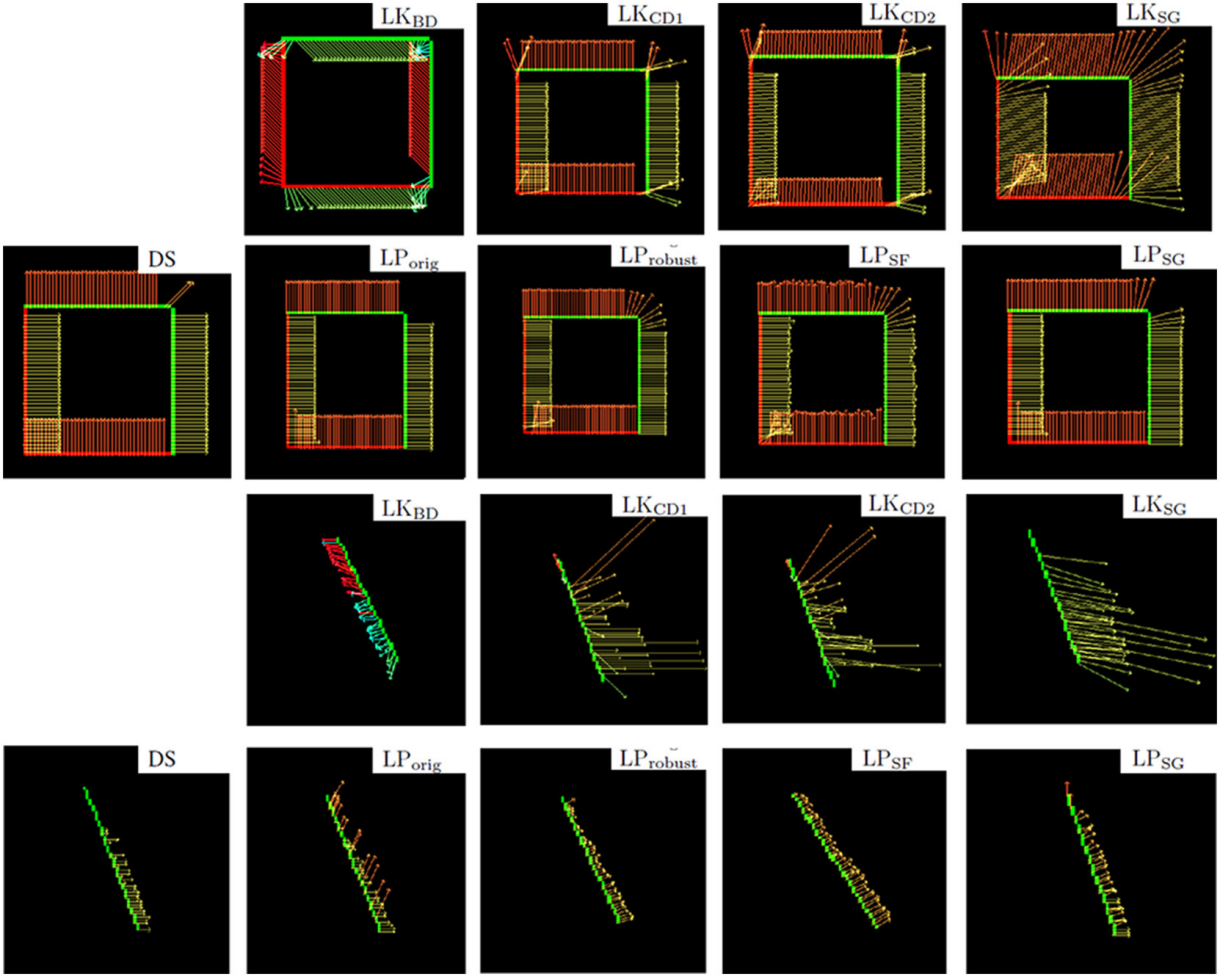
**First two rows: Flow fields computed with all nine methods, of the synthetic translating square sample**. Last two rows: rotating bar. In this and the following figures, vectors are colored according to the angle of the motion event for better readability. The method *LK*_*BD*_ fails to detect the correct direction of motion in these synthesized samples due to the asymmetry of the backward finite difference approximation (discussed in Section 2.1.2).

Figure 3 illustrates the flow fields produced by all nine methods for the synthesized samples translating square and rotating bar.

**Figure 4:**
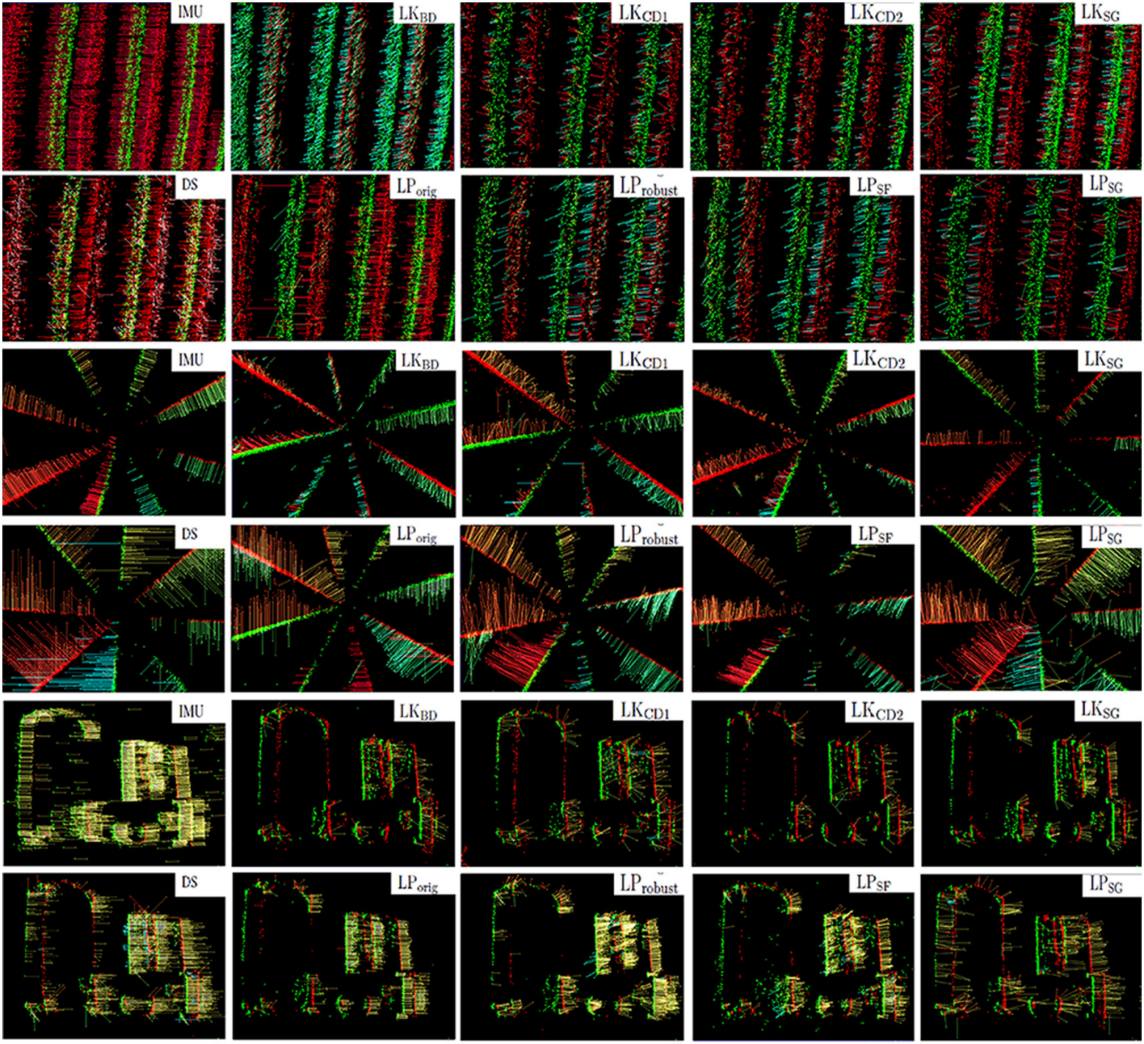
**Ground truth from IMU and flow fields computed with the nine motion flow algorithms, of the three real samples: translating sinusoid, rotating disk and translating boxes**.

In Figure 4, the flow fields computed by the nine methods are compared with ground truth based on camera rotation (IMU), for the translating sinusoid, rotating disk and translating boxes sample.

The event-density reported in the supplementary material signifies the fraction of events that passed the filter with valid flow vector attached. As mentioned in Section 2.5, the algorithms contain various tests to improve the results and discard outliers. These tests have a noticeable effect on computation time: For instance, the refractory period skips computation of affected events entirely. At the same time, the motion field is thinned out. The event-density is a direct indicator of these two effects.

### 3.1. IMU integration: Verification of ground truth

Table 1 summarizes three rudimentary tests to check proper functioning of the IMU flow. The horizontal speed of the translating sinusoid and the box scene as well as the angular velocity of the rotating disk was measured and compared to the IMU flow estimate. This manual measurement consisted in tracing a patch of the moving object for a certain distance and calculating the speed by the time of flight. The main uncertainty with this method lay in tracing the exact location and is estimated to be one pixel.

**Table 1:** **Comparison of ground truth with manual measurements of translational / rotational object motion for the three real samples**.

**Sample**	**Manually**	**IMU**	**Rel. error**
translSin	31 px/s	30.05 px/s	3%
rotDisk	0.52 1/s	0.57 1/s	8.8%
translBox	26 px/s	24.5 px/s	6.6%

*The relative error is given with respect to the manual measurement*.

Without IMU offset correction, the IMU constantly outputs some non-zero gyro data that results in flow vectors even when the sensor is at rest. These offsets are computed during stationary camera conditions by averaging several hundred samples and then subtracting these offsets from subsequent readings. This subtraction reduces but not completely removes the error. The remaining non-zero flow vector at rest is much less than a pixel per second and the resulting angular error is only a small fraction of a degree.

See Figure 4 for examples of the motion field flow vectors produced by the IMU-based method.

### 3.2. Processing time

Table 2 summarizes the processing time measurements for each of the 10 methods (including IMU flow) for the translating boxes sample. The fastest variant of each method is highlighted in bold. Table 3 shows the approximate floating point operation count for each of the methods.

**Table 2:** **Average processing time per event in microseconds with standard deviation**.

**PT [μs]**	**Mean**	**SD**
IMU	**0.38**	0.04
LK_BD_	5.32	0.21
LK_CD1_	5.06	0.30
LK_CD2_	8.99	0.29
LK_SG_	**3.13**	0.27
LP_orig_	4.35	0.31
LP_robust_	4.51	0.28
LP_SF_	0.70	0.13
LP_SG_	**0.58**	0.03
DS	**0.36**	0.02

**Table 3:** **Approximate number of floating point operations needed to calculate the motion vector of one event, for each algorithm**.

	**FLOPs**
IMU	100
LK_BD_	1857
LK_CD1_	1957
LK_CD2_	2557
LK_SG_	1372
LP_orig_	1827
LP_robust_	1840
LP_SF_	1359
LP_SG_	980
DS	1096

### 3.3. Accuracy

In Table 4 we summarize the angular accuracy of the nine methods for all samples; Table 5 shows the relative endpoint error. The absolute endpoint error as well as robustness measures for angle and endpoint error are provided in the supplementary material. Given that the accuracy of each algorithm varies somewhat across the dataset, it is difficult to detect a trend or identify the best performing method. Note however that among the LucasKanade variants, the Savitzky-Golay filter scores best in terms of angular accuracy on 4 out of 5 samples, as does the DirectionSelective filter. The Savitzky-Golay variation of the LocalPlanes method computes accurate angles but struggles with the correct vector magnitude on three of the samples. This behavior is discussed further in Section 4.2.

**Table 4:** **Average angular error in degrees with standard deviations**.

**AAE [°]**	**translSqu**	**rotBar**	**translSin**	**rotDisk**	**translBoxes**
LK_BD_	135.77 ± 31.45	73.24 ± 56.52	20.35 ± 16.46	51.71 ± 45.47	108.07 ± 28.67
LK_CD1_	**7.05** ± 20.05	29.68 ± 20.54	21.72 ± 35.31	19.93 ± 21.35	28.98 ± 31.27
LK_CD2_	9.38 ± 19.31	32.85 ± 21.17	13.33 ± 15.18	18.72 ± 19.00	36.07 ± 36.69
LK_SG_	11.48 ± 8.80	**16.75** ± 8.26	**9.56** ± 15.90	**6.37** ± 6.53	**12.13** ± 19.84
LP_orig_	**0.00** ± 0.00	17.54 ± 21.56	38.93 ± 61.85	28.06 ± 33.39	9.78 ± 32.96
LP_robust_	**0.00** ± 0.00	9.56 ± 29.97	37.72 ± 55.76	22.30 ± 32.70	9.46 ± 22.67
LP_SF_	2.39 ± 8.98	**6.81** ± 24.34	43.99 ± 48.52	23.39 ± 32.12	**9.27** ± 13.11
LP_SG_	**0.00** ± 0.00	8.72 ± 17.92	**28.39** ± 42.78	**18.77** ± 31.74	13.96 ± 23.62
DS	**0.63** ± 5.28	**6.88** ± 7.18	32.82 ± 56.67	**16.62** ± 20.36	**12.01** ± 31.91

Values in bold face are smallest error for each algorithm.

**Table 5:** **Relative average endpoint error in percent (normalized with respect to the magnitude of the ground truth velocity), with standard deviations**.

**AAE_rel_[%]**	**translSqu**	**rotBar**	**translSin**	**rotDisk**	**translBoxes**
LK_BD_	123.43 ± 11.04	414.95 ± 767.50	57.85 ± 16.37	76.65 ± 31.87	116.95 ± 15.57
LK_CD1_	**12.94** ± 36.16	197.47 ± 386.69	57.16 ± 25.55	54.46 ± 34.89	58.13 ± 28.86
LK_CD2_	32.87 ± 24.43	**183.69** ± 276.91	37.54 ± 21.52	64.53 ± 24.52	72.54 ± 27.70
LK_SG_	65.08 ± 21.08	326.77 ± 253.31	**32.50** ± 26.98	**49.83** ± 27.64	**35.62** ± 24.68
LP_orig_	**0.00** ± 0.00	175.08 ± 460.93	62.82 ± 48.67	60.71 ± 61.76	37.93 ± 35.15
LP_robust_	**0.00** ± 0.00	91.61 ± 278.97	59.45 ± 37.49	**50.57** ± 37.08	36.13 ± 27.80
LP_SF_	6.41 ± 15.13	**39.08** ± 63.84	69.62 ± 33.17	58.99 ± 37.87	**33.49** ± 18.01
LP_SG_	**0.00** ± 0.00	114.56 ± 341.18	**54.41** ± 51.27	78.02 ± 281.03	66.74 ± 44.78
DS	**1.06** ± 8.99	**40.37** ± 54.65	62.92 ± 60.50	**44.18** ± 34.91	**30.73** ± 34.06

## 4. Discussion

This study compares accuracy and processing time of nine event-based optical flow algorithms. A direct comparison with frame-based methods is possible by applying them on the frames included in this dataset (which has not been done yet). In order to use existing frame-based benchmarks (e.g., on the Middlebury database), (Barranco et al., 2015) created synthetic events by simulating 50,000 frames for every two frames and interpolating frame-by-frame changes in intensity. This was not feasible here because our implementations compute normal flow, whereas the Middlebury ground truth provides 2D flow. However, the comparison of event- and frame-based algorithms running on seven samples of the Middlebury dataset, as done by Barranco et al., offers at least some rough indicator for the performance of the methods presented here with respect to previous results. Their novel phase-based motion estimation achieves relative AEE between 16.6 and 42.5% for seven of the Middlebury sequences; the accuracy of the then best-performing frame-based method ranges between 1.6 and 46.6% endpoint error on the same samples. Several of the methods here (evaluated on the dataset here) perform within this range.

### 4.1. Processing time

The fastest method was the direction selective filter with 0.36 μs per event, where no extensive linear algebra is performed. The next fastest method is the Savitzky-Golay-filter variant of the Local Plane Fit method, which with 0.58 μs is about eight times faster than the original version because computing the fitting parameters does not involve solving a system of equations. *LP*_*SF*_ is almost as fast because it does not repeatedly improve the initial plane fit. However, it has to solve a linear system of equations once per event with linear least squares. The standard Lucas-Kanade methods take between 5 and 9 μs per event, while its Savitzky-Golay variant ranges at 3.13 μs. Calculating the motion field with the IMU data is very fast (0.38 μs per event) because the transformation of the event-coordinates (rotation and translation) is not costly, and the rotation matrix and translation vector has to be computed only about every millisecond when an update from the IMU comes in.

All methods take less than 10 μs to calculate the motion vector of a single event; DS, IMU and *LP*_*SG*_ take less than one μs per event. Thus, they all run in real-time on contemporary PCs^17^ if the event-rate does not exceed 1e5 events per second (eps), or 4000 events per frame with a frame-rate of 25 fps. The mean event rate of our rotating disk sample is 1e5, and 3e5 eps for the rather dense translating sinusoid, which can still be processed in real-time by four of the methods. Natural input even from data recorded by DAVIS240 cameras on moving platforms seldom exceeds 1e6 eps. In case it does, the sequence would have to be processed by *LP*_*SG*_ or DS, or subsampled to reduce event density and make it suitable for slower algorithms. Subsampling is usually unproblematic in global motion estimation (e.g., for motion stabilization), but might impede local flow estimation (e.g., object tracking).

The algorithms are implemented in pure Java but became much faster by relying on just-in-time optimization and performing most linear algebra explicitly in pure Java rather than by using numerical libraries like jama or jblas. As pointed out by Barranco et al. (2014), event-based optical flow algorithms could potentially be parallelized in graphics processing units or pipelined in field-programmable gate arrays, although the challenges of applying what are naturally SIMD hardware to causal and time-ordered event-based sensor data remain to be addressed. A solution based on parallel hardware units that process each pixel independently is not economical because most such units would be idle most of the time, so developing hardware units that can perform the necessary computations on the memory that stores either timestamps, histograms, or orientation features is not straightforward. If the units pipeline the operations to deal with a queue of events, then they must deal with memory bus contention to access the large timestamp arrays. If the units have dedicated blocks of memory to deal with blocks of pixels, then they must handle the block edges.

### 4.2. Accuracy

Methods that performed well in terms of angular error were the Savitzky-Golay variants of the Lucas-Kanade and Local Plane Fit algorithm, and - surprisingly, given its limitation through the quantized angle - the DirectionSelective filter. The derivative estimation with central finite differences instead of backward differences clearly improves finding the correct direction. Similarly, computing the plane fitting parameters with robust Equation (9) improves performance of the original version, most distinctly in the rotating bar and disk samples. There, the original method suffers from angle quantization, as outlined in Section 2.1.3. Evaluation of the endpoint error reveals a similar picture, though the LocalPlane Savitzky-Golay variant is not as good as for the AAE.

The standard deviation is of the same order as the mean, for all methods and samples. There are at least two possible explanations. First, the estimated flow vectors stray around the mean due to the noisy event-structure at contours. Second, a substantial portion of vectors point quite oppositely to the true direction of motion (see Section 4.2.1 for a discussion of this phenomenon). Together with the knowledge that the variation is a dispersion measure sensitive to outliers (because distances from the mean are squared), this accounts for the large standard deviation. A more robust statistic would be the median absolute deviation around the median, or the mean absolute deviation around the mean, which are more resilient to outliers.

#### 4.2.1. Lucas-Kanade

**Figure 5:**
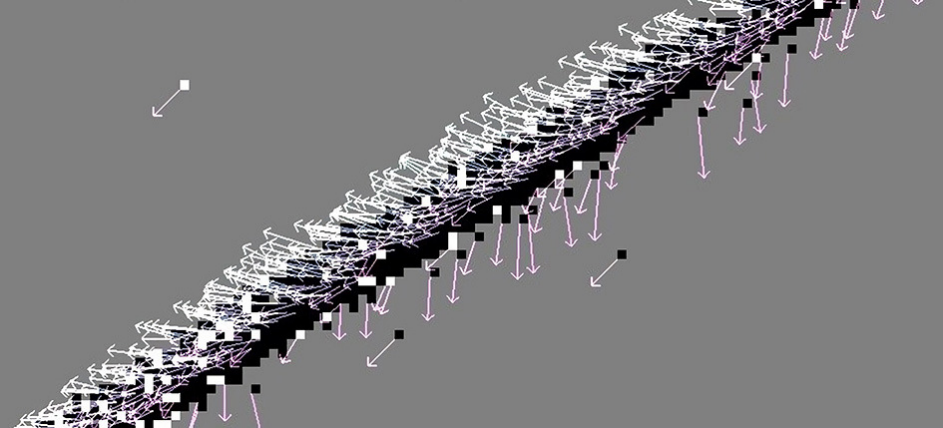
**The Lucas-Kanade method where the intensity derivative is approximated by backward finite differences (kernel [−1, 1] and [1, −1]^*T*^)**. Clearly visible is the bias toward left and down; the true direction of motion is perpendicular to the contour orientation.

**Figure 6:**
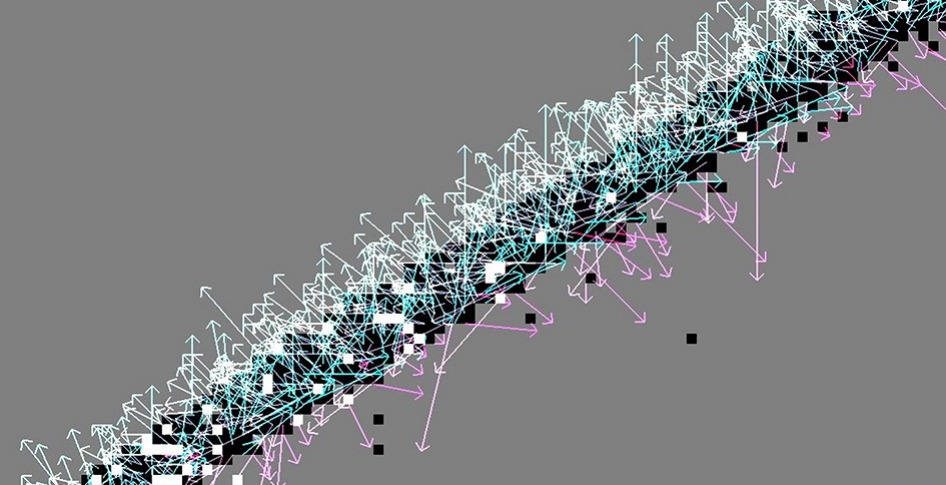
**Using a central finite difference derivation removes the bias of the backward finite difference method: The flow vectors stray around the true direction of motion (with large variance due to the noisy contour)**.

A vital part of the Lucas-Kanade variants is the estimation of intensity derivatives using finite differences. This is a relatively crude form of numerical differentiation given the highly discontinuous and noisy intensity (event frequency) function. Comparing Figure 5 with Figure 6 illustrates how the backward finite difference is biased toward the left and down, resulting in large errors (The true direction of motion is orthogonal to the contour orientation). This bias is lifted by using central finite differences (see Figure 6 with flow from method *LK*_*CD*1_). A second order (8-point) central difference derivative (*LK*_*CD*2_) does not pay off significantly. While for some samples the error is reduced by a few percent, using a higher order in fact increases the angular error for other samples by up to 10%. The derivative is more accurate, but also more susceptible to noise.

The consistent use of second temporal derivatives on the RHS of Equation (5) is problematic because of the small number of events in a neighborhood, as outlined by the authors proposing this modification (Brosch et al., 2015). The effect is similar to that of *LK*_*CD*2_: Depending on the image sequence, it increases the error by up to 10% because noise is amplified. Apart from its influence on accuracy, the second temporal derivative increases the processing time by about 50% because in our implementation we now need to loop over the neighborhood twice to find the number of events at each pixel location in two different time windows. Note that we did not explicitly include a comparison between the use of first and second temporal derivative in the tables in Section 3; all Lucas-Kanade methods there employ the second temporal derivative.

**Figure 7:**
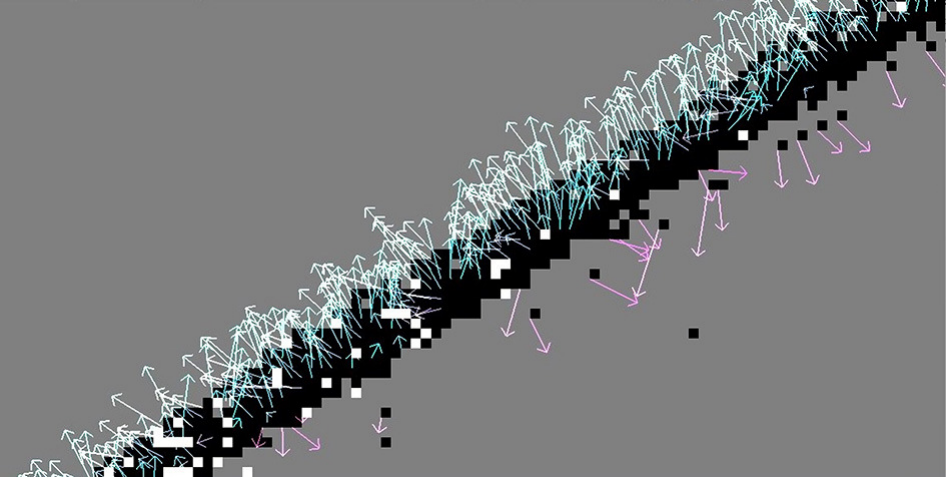
**A refractory period of 15 ms is added to the otherwise identical situation of Figure 6**. Events in the back of the edge are filtered out, which decreases the number of outliers (AAE reduced by 30%).

As visible in Figure 6, high angular errors (flow vectors oppositely to the true direction) occur at the back side of a moving edge. Recall that the motion vector points along the gradient of the intensity function, i.e., from sites with higher event frequency to those with less. At the front of the edge this produces correct results. But an event at the back of the moving edge will see the opposite gradient: When looking at the event histogram, there are steadily new events toward the direction of motion, and no more events coming from the homogeneous center of the moving object, so the flow vector will point the wrong way. This effect can be reduced by including a refractory period τ_*rf*_, which allows skipping the calculation of an event's motion flow vector when the pixel has fired its last event within τ_*rf*_.^18^ The pixels in the back of the edge have most likely fired recently (within the last 10–100 ms), so by setting τ_*rf*_ = 15 ms most of them are excluded. In Figure 7, the AAE is thus reduced by 30% compared to Figure 6. Of course there are some pixels that have not fired before, even though the edge passed over them; they will still produce incorrect results. This refractory period must be adjusted appropriately for the dynamics of any particular application.

**Figure 8:**
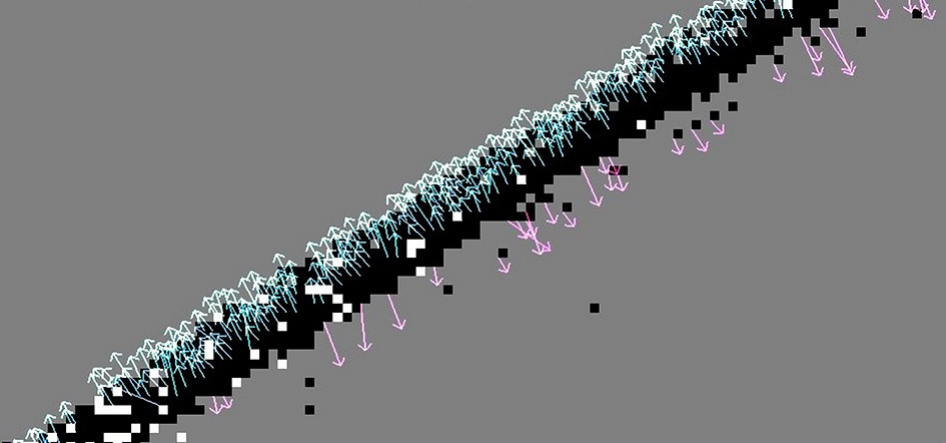
**Lucas-Kanade with a two-dimensional linear Savitzky-Golay filter to smooth the event frequency function before numerical differentiation**. Note the reduced angular error compared to the method in Figure 7.

Figure 8 shows how another significant improvement is achieved by convolving the noisy event-frequency function with a two-dimensional Savitzky-Golay filter as described in Section 2.1.2.

All Lucas-Kanade variants display considerably higher AEE on the synthetic samples translSqu and rotBar. This is due to fact that a moving edge e.g., in translSqu consists of a line of single events with equal timestamps stepping to the adjacent pixel location every 50 ms. The event rate function on which the derivatives are computed thus contains only a constant one event per time bin, regardless of how fast the edge is moving. (In a real sample, a faster moving edge would accumulate less events at a given pixel than a slow one.) These artificial samples thus turn out to be inadequate for Lucas-Kanade methods.

#### 4.2.2. Local plane fit

Local area methods that are causally event-based know nothing about what lies ahead of a leading edge. This is not a problem in Lucas-Kanade-like methods above, where the flow vectors basically point from regions of higher event-rate to those with less. Local plane fit methods however operate on the surface of most recent events, where a large portion of the timestamp data points may lie quite far away (stemming from previous motions of other objects), or may have never been set at all (e.g., when looking from the past to the future side of a moving edge). This makes estimation of fitting parameters susceptible to noise and, due to sparse distribution of valid events, often impossible.

The local plane fit as well as Lucas-Kanade methods perform badly in the presence of texture: A contour moving over a textured background produces a varying number of events along the edge and over time because the contrast between edge and background changes due to the texture. This can be dealt with by applying Gabor-filtering and using a phase-based instead of gradient-based method (Barranco et al., 2015). The error is shown to be reduced by 30%. However, the algorithm does not run in real-time.

Local plane fits become unreliable when the edge is broad. Consider an edge moving to the left as in the translating sinusoid sample. The pixels in the leftmost column of the edge can fire at almost the same time as pixels in the adjacent column, not because the edge moved so fast, but because its contrast changes continuously. This causes large variations in the estimated speed of the flow and a general overestimation of speed in the Local Planes methods. The AEE reflecting this can be reduced by a factor of 2–3 by prefiltering the events with a refractory period^19^ of τ_rf_≈100*ms*. This refractory period prevents updating the timestamps of the surface of most recent events if the pixel has fired an event within the refractory period. This reduces the effect of speed errors due to synchronous events in a broad edge. This refractory period is different from the refractory period applied to the LucasKanade methods, which skips computing the flow vector but nevertheless stores the timestamps of events that fall within the refractory period. This other refractory period contributes to a more robust estimate of the derivatives of the event-rate function.

A large neighborhood increases the chances that the surface of active events contains structure that is not well approximated by a local plane, while a small neighborhood runs the risk of containing not enough valid events for a robust fit. Other contributing factors are multiple motions or transparent motion stimuli (Brosch et al., 2015). The problem of textured contours is addressed comprehensively in Barranco et al. (2014).

#### 4.2.3. Direction selective

Recall that this method computes the flow vectors by first determining the orientation of a contour, and then looks for past orientation events perpendicular to it. The speed is then the pixel distance divided by the average time-difference to this past event over a search distance (typically 5 pixels). Angular errors are introduced in the first stage, endpoint (speed) errors in the second stage. Thus, like the other methods, the direction selective flow computation suffers from noisy and uneven contours, because then the local orientations of small contour-patches vary, and with it the direction of normal velocity. But even if the algorithm found the correct orientation (low angular error), the magnitude of the computed velocity (endpoint error) may vary by a factor of 100. This error is due to the fact that the events *along the direction of motion* are by no means homogeneously distributed. For an object moving with constant velocity (e.g., the translating sinusoid), a certain pixel may fire very quickly after an adjacent pixel (resulting in high speed), while another pixel of the same edge fires much later than its neighbor (resulting in low speed). A test stage that filters out speeds far above average helps remove this effect in large part.

Another source of error is the quantization of vector direction in bins of 45°. In our dataset, the DS filter is favored by the use of pure pan and tilt movements and this bias should be taken into account by users who desire continuous angle motion vectors.

A different event-based approach to motion estimation using direction-selective filters has been taken recently by Brosch et al. (2015). They use biologically inspired spatio-temporal filter-banks to detect the orientation of an edge. This even allows distinguishing multiple directions at a single location (e.g., for transparency). They also include a response normalization stage that reduces motion ambiguity: The aperture problem faced by any local gradient-based optical flow algorithm is tackled by inhibiting normal flow components. Using a dynamic neuron model, the normal components in the center of a contour are strongly reduced compared to the ones at corners, thus biasing the flow vector histogram toward the true direction of motion. However, this rather sophisticated technique is currently too expensive for real-time application. A way to handle the computations more efficiently could be to use spiking neural network hardware on a neuromorphic processor together with the event-generating dynamic vision sensor at the front end.

#### 4.2.4. Ground truth from IMU

The rotation rates used to compute ground truth are updated with 2.5 kHz. This quantized time is a potential error source in fast motion. Considering a pure rotation about the z-axis (roll), each image point describes an arc, but our method approximates this segment with a straight line, introducing an error in speed and direction. However, calculating the angle between the tangent to the endpoint of the circular arc and the secant line approximating this arc, it becomes clear that this error is not significant in most situations. For instance, if the sensor turns with less than 1.39 rounds per second, the error in direction due to a finite sample rate stays below 0.1 degree. Nevertheless, a way to make this source of ground truth applicable even to very fast rotations or slower IMU update rates, one could use the rotational velocities directly instead of integrating to get the rotation angle. This would require a rotation about the focal point and a coordinate transform from the IMU pose to the focal point. The motion field then follows directly from the well-known relations between the scene and the sensor.

## 5. Conclusion

In this report we compare nine basic algorithms that compute optical flow based on address-events from a neuromorphic dynamic vision sensor. A tenth method is presented that allows estimation of the motion field in real time using camera rotation rates obtained from an inertial measurement unit mounted on the camera. Based on this ground truth, the nine methods are tested for three real sequences that seem simplistic but nevertheless reveal fundamental challenges of event-based motion flow, namely noisy and fissured contours.

Six of the methods presented above are variants of the Lucas-Kanade algorithm (Benosman et al., 2012) and the Local Plane Fit (Benosman et al., 2014). *LK*_*BD*_, *LK*_*CD*1_, and *LK*_*CD*2_ evaluate the performance of three finite difference methods and provide evidence of how critical the numeric derivative approximation is. *LP*_*robust*_ and the consistent use of a second temporal derivative in the Lucas-Kanade methods were suggested by Brosch et al. (2015). The former carries a clear improvement especially in terms of angular accuracy because the numerical problems of inverting small derivatives are avoided. The latter does improve the results on some samples but fails to do so on others, because the second derivative is susceptible to the coarse and unstable nature of the intensity function in AER. The Savitzky-Golay variants were introduced in an attempt to smooth out this noisy event cloud. These two methods not only speed up processing time, but show improved angular accuracy.

All methods run in real-time if the event-rate is below 1e5 events per second; DS, IMU and *LP*_*SG*_ are able to handle 1e6 events per second, which is rarely exceeded by dense naturalistic input. Compared to the original Lucas-Kanade (Benosman et al., 2012) and Local Plane Fit (Benosman et al., 2014) algorithm, our implementations^20^ *LK*_*BD*_ and *LP*_*orig*_ reduced the computational cost by 26 and 29% respectively.

We also discuss the error statistics used so far and suggest a purely angular measure between flow vectors in the image plane. This serves to lessen the bias inherent in the conventional average angular error and to separate a deviation in direction from speed error, to better expose aspects requiring improvement.

With the IMU-integration we establish a new performance measure to compare various motion flow algorithms in situations where the direction normal to edges is known.

The data set of consisting of a mixture of DVS and DAVIS data collected for this study has been shared to provide a baseline for future comparison^21^. All the algorithms developed here are open-sourced in the jAER project, in the Java package ch.unizh.ini.jaer.projects.rbodo.opticalflow^22^. The supplementary data to this paper includes images of the experimental setup and the real data samples. Videos (in the database repository) of several of the algorithms show how they behave dynamically for some of the test cases studied here. Complete result tables compare the algorithm accuracy and cost for the different test cases, including robustness measures (like the percentage of flow vectors with angle errors above 3°).

It is easy to record natural data for which all of the described algorithms fail rather dramatically, but further pursuit of accurate event-based methods with DVSs is worthwhile considering that this neuromorphic hardware fits many of the challenging demands of real-time applications in terms of low power consumption, small response latency, and efficient use of hardware resources. One implication of this report is that accuracy can be improved with modifications that actually reduce computation time significantly. We expect that use of DAVIS and ATIS (Posch et al., 2011) intensity information and the development of efficient area-matching-based rather than gradient based methods will further enhance accuracy in the future.

## Author contributions

BR developed the algorithms, collected and analyzed the data, and wrote the body of the paper. TD contributed guidance and writing.

### Conflict of interest statement

The authors declare that the research was conducted in the absence of any commercial or financial relationships that could be construed as a potential conflict of interest.

## Footnotes

^1^*Middlebury Optical Flow*, http://vision.middlebury.edu/flow/.

^2^*jAER Open Source Project*, http://www.jaerproject.org.

^3^SimpleOrientationFilter source code.

^4^LukasKanadeFlow source code.

^5^This assumption does not hold if the neighborhood covers motion boundaries, which are admittedly the most interesting regions for determining structure from motion. Because most information in event-based methods comes from contours, this may well be a significant error source that our dataset does not cover well because of its basis in the motion field caused by camera rotation. However, events at an edge typically span a width of several pixels when accumulated over the time window set for flow computation, so except for the outermost events the assumption will hold.

^6^The parameter *thr* in the jAER implementation *LucasKanadeFlow*.

^7^LocalPlanesFlow source code.

^8^*OriginalLP* method in LocalPlanesFlow jAER class.

^9^*RobustLP* method in LocalPlanesFlow jAER class.

^10^*SingleFit* method in LocalPlanesFlow jAER filter.

^11^*LinearSavistskyGolay* method in LocalPlanesFlow jAER class.

^12^IMUFlow source code; uses class AbstractMotionFlowIMU in method calculateImuFlow.

^13^inilabs.com

^14^This dataset is hosted at http://sensors.ini.uzh.ch/databases.html.

^15^We write DVS to mean the DAVIS240c throughout this paper, because only the DVS events from the DAVIS240c were used. However, the dataset includes synchronous frames.

^16^Matlab scripts folder in jAER. The synthetic data is exported from matlab using the script saveaerdat.m.

^17^Which however burn on the order of 100 W.

^18^Besides the positive effect on accuracy discussed here, the refractory period can also be used to thin down the motion field for better visibility and computation speed. This does not affect the motion flow calculation of other events: If the refractory period test returns true, i.e., the filter is told to skip this event and move on to the next, the filter nevertheless memorizes the event. Thus, it is part of the neighborhood of the next event close by whose motion flow is calculated.

^19^RefractoryFilter source code.

^20^http://sensors.ini.uzh.ch/databases.html

^21^in pure Java rather than C++ as Benosman et al. (2012), both with dual core processors @ 2.4 GHz.

^22^*jAER Open Source Project*, http://www.jaerproject.org.
